# Rapid Microbial Dynamics in Response to an Induced Wetting Event in Antarctic Dry Valley Soils

**DOI:** 10.3389/fmicb.2019.00621

**Published:** 2019-04-04

**Authors:** Thomas D. Niederberger, Eric M. Bottos, Jill A. Sohm, Troy Gunderson, Alex Parker, Kathryn J. Coyne, Douglas G. Capone, Edward J. Carpenter, Stephen Craig Cary

**Affiliations:** ^1^College Earth, Ocean, and Environment, University of Delaware, Lewes, DE, United States; ^2^International Centre for Terrestrial Antarctic Research, School of Science, University of Waikato, Hamilton, New Zealand; ^3^Department of Biological Sciences, Thompson Rivers University, Kamloops, BC, Canada; ^4^Department of Biological Sciences, Wrigley Institute for Environmental Studies, University of Southern California, Los Angeles, CA, United States; ^5^Romberg Tiburon Center, San Francisco State University, Tiburon, CA, United States

**Keywords:** Dry Valleys, climate change, cyanobacteria, DNA fingerprinting, wetting

## Abstract

The cold deserts of the McMurdo Dry Valleys (MDV), Antarctica, host a high level of microbial diversity. Microbial composition and biomass in arid vs. ephemerally wetted regions are distinctly different, with wetted communities representing hot spots of microbial activity that are important zones for biogeochemical cycling. While climatic change is likely to cause wetting in areas not historically subject to wetting events, the responses of microorganisms inhabiting arid soils to water addition is unknown. The purpose of this study was to observe how an associated, yet non-wetted microbial community responds to an extended addition of water. Water from a stream was diverted to an adjacent area of arid soil with changes in microbial composition and activities monitored via molecular and biochemical methods over 7 weeks. The frequency of genetic signatures related to both prokaryotic and eukaryotic organisms adapted to MDV aquatic conditions increased during the limited 7 week period, indicating that the soil community was transitioning into a typical “high-productivity” MDV community. This work is consistent with current predictions that MDV microbial communities in arid regions are highly sensitive to climate change, and further supports the notion that changes in community structure and associated biogeochemical cycling may occur much more rapidly than predicted.

## Introduction

The McMurdo Dry Valley (MDV) system of Antarctica represents the largest ice-free region of the Antarctic continent (Levy, 2013). The combination of extensive glacial scouring, intense katabatic winds and extremely low precipitation rates (Keys, 1980; Doran et al., 2002) make them arguably the oldest, coldest and driest deserts on Earth. The soils within the MDV typically contain low organic matter, high salt and pH levels, and water content below 2% (reviewed by Cary et al., 2010). These soils support a very simple biological trophic structure that is dominated by microorganisms and is characterized by a lack of vascular plants and limited invertebrate taxa (Franzmann, 1996; Wynn-Williams, 1996; Moorhead et al., 2003; Adams et al., 2006). Early culture-based microbial studies (Cameron et al., 1968) suggested that the soil bacterial diversity and abundance in these cold desert areas was extremely low, yet the application of molecular-based techniques (Smith et al., 2006; Niederberger et al., 2008; Babalola et al., 2009; Pointing et al., 2009; Lee et al., 2012) has now revealed a higher than expected level of microbial diversity (Cary et al., 2010; Bottos et al., 2014b), especially considering the extreme nature of the system.

The bulk mineral soils of the MDV are considered extremely arid, however, warm summer temperatures and increased solar radiation promote the melting of exposed surfaces of glaciers resulting in the formation of ephemeral melt-water streams that flow periodically for 4–12 weeks (McKnight et al., 1999, 2007). Through summer-melt, the wetted soils adjacent to the glacial melt-water streams, defined as the “wetted zone,” and the wetted moat soils surrounding lake systems can extend up to several meters on either side of the water source and constitute hotspots for microbial life. These areas are rapidly transformed into important zones for biogeochemical cycling (McKnight et al., 1999, 2007; Gooseff et al., 2003; Niederberger et al., 2012). Biological crust communities (thin microbial mats) form in these annually wetted zones, surviving the winter months in a desiccated state before re-activation through summer melt-water hydration. As a result, these communities can form large concentrations of responsive biomass even under extreme *in situ* environmental conditions (Hawes et al., 1992; McKnight et al., 1999, 2007).

Oases of high microbial productivity may represent the keystone of ecosystem function for the entire MDV system. Products from these microbial activities feed more complex food webs and through aeolian-based transportation, mat material may represent an important carbon source to an otherwise highly oligotrophic soil system (Hopkins et al., 2006a,b; Barrett et al., 2007; Zeglin et al., 2011). Although several studies have consequently focused on these ephemerally wetted communities and accompanying nutrient cycling (McKnight and Tate, 1997; Runkel et al., 1998; Maurice et al., 2002; Gooseff et al., 2004; Zeglin et al., 2011; Niederberger et al., 2012), there has been only a single study describing events during the rewetting of a relic stream bed (McKnight et al., 2007).

Microbial communities in the MDV soils are highly sensitive to climate change (Nielsen and Wall, 2013) and it is predicted that the continent will continue to become warmer and wetter (Bracegirdle et al., 2008). As a consequence of recent warming trends, most MDV lakes have risen more than 1 m since records began in 1986 (McKnight et al., 1999) with recent rapid topographic changes observed in the valleys due to wetting events (Fountain et al., 2014). While the microbial mats in wetted zones appear to have formed over many wet/dry cycles, these recent climatic changes are likely to cause wetting in areas that have not historically been subject to wetting events. Although studies have examined changes in microbial community composition upon addition of water (e.g., Van Horn et al., 2014; Buelow et al., 2016), little is known about community response within archetypal arid MDV soils to a “natural” wetting event from rising lakes or changes in topography, or how these responses lead to the development of microbial-mat communities that possibly drive increased biodiversity.

The purpose of this study was to observe how an arid microbial community responds, in both its composition and structure, to the addition of water. While the addition of filtered water would demonstrate a response within the resident community, a more realistic approach requires the diversion of water with its resident biological community intact. Changes in the community composition and structure should reflect the interactions between the resident “arid-adapted” community, cryptobiotic species which are reactivated upon addition of water (McKnight et al., 2007), and “wet-adapted” members of the biological community recruited from the diverted water source. In order to achieve this, we diverted a portion of the water from a small MDV stream to an adjacent area of arid soil and monitored microbial community dynamics and activities via molecular- and biochemical-based methods over an entire summer season. This experiment provides essential detail about the time course of response of MDV microbial communities to natural wetting events, without permanently altering stream flow. This investigation greatly adds to the few comprehensive investigations of microbial community dynamics and metabolic activities in relation to the physicochemical environment in polar ecosystems (e.g., McKnight et al., 2007; Pointing et al., 2009; Tiao et al., 2012; Sokol et al., 2013; Stanish et al., 2013; Van Horn et al., 2013, 2016; Geyer et al., 2014). Moreover, results from this work will contribute to our understanding of future climate-change impacts on MDV ecosystems. Our results demonstrate significant changes in community structure in arid MDV soils due to the sudden availability of water and recruitment of wet-adapted microbial species, and confirm that MDV soil communities respond rapidly to these events. This information adds to the recent reports in the literature (Tiao et al., 2012; Van Horn et al., 2014, 2016; Buelow et al., 2016) that indigenous MDV soil communities have the potential to undergo surprisingly rapid changes due to changing environmental conditions.

## Materials and Methods

**Figure 1:**
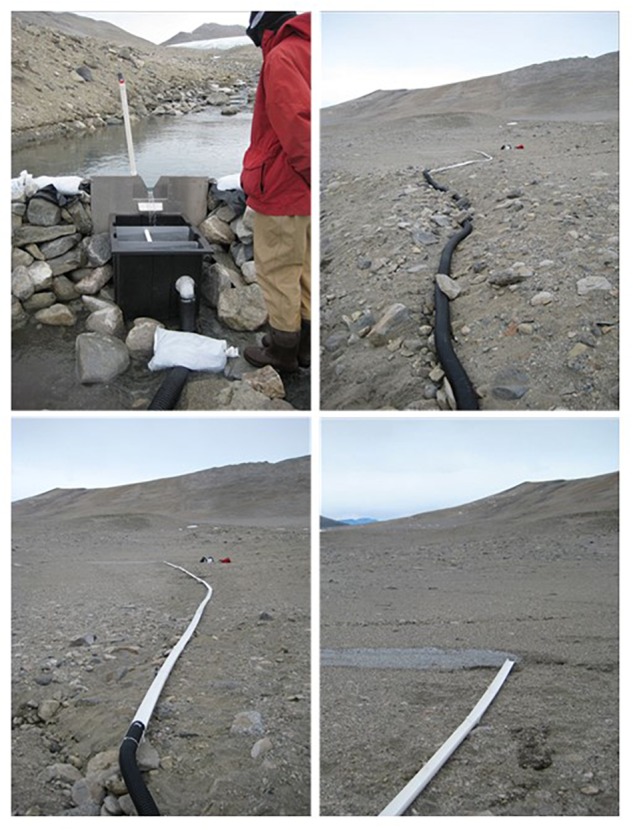
Images of the dam, weir, and tubing.

The Adams and Miers Glaciers are located at the upper (Western) part of Miers Valley and have glacial run-off streams that converge and flow into the central Miers Lake. For this experiment, a portion of the Adams Glacial run-off stream was diverted to dry soil (moisture content typically < 5%; Niederberger et al., 2015). The diversion (Figure 1) was constructed using alluvium found in or next to the stream itself and sandbags filled with stream derived mineral gravel and the dam “water proofed” using black plastic sheeting. The stream flow was diverted through a V-notch weir (located at S78°05.947, E163°46.275) into a small tank, then through a 110 mm diameter black plastic tubing and into deep plastic open guttering that directed the water to a nearby dry area of mineral soil (S78°05.960, E163°46.400). See Figure 1 for images of the dam, weir and tubing. The length of the pipe and channeling restricted the wetted area to ∼100 m. The experiment commenced on the 4th of December, 2009 and ran for 7 weeks (Table 1). The flow of water was irregular due to multiple silting and freezing events. An initial composite sample (T0) was collected by combining samples from 1, 4, and 12 m downstream from the outflow and before the start of the experiment. Samples were then taken from soils at 1, 6, 12, 24, and 72 h from 1 m downstream from the diverted water flow, and at 24 and 72 h from 4 m downstream after the start of the manipulation. Weekly samples were also collected from 4 and 12 m downstream from the outflow. Composite soil samples, in which several individual samples were mixed from each site to reduce patchiness, were collected using a sterile spatula in Whirl-Pak^®^ bags. In some instances, samples were not collected due to logistical constraints. To aid recovery, after the experiment the area was returned to its natural state as best as possible with the aid of photographs taken prior to the diversion experiment.

**Table 1:** Soil samples collected over the time course of the wetting experiment and associated data.

Sample time^a^	Location (m)	pH	NO_x_^b^ (μM)	Silicate (μM)	Orthophosphate (μM)	Ammonium (μM)	DIC (μmol C L^-1^)	Thymidine (μg C/cc/h)	Chl *a*^c^ (μg/cc)	Cell counts
0		6.20	3.8	9.0	0.1	0.53	479	0.2337	0.051	4.646E+05
1 h	1	7.14	6.0	23.3	0.3	2.00	548	NA	0.019	4.523E+05
6 h	1	7.71	NA	NA	NA	NA	NA	NA	NA	NA
12 h	1	7.68	NA	NA	NA	NA	NA	NA	NA	NA
24 h	1	6.96	5.2	6.4	0.0	1.57	NA	0.1069	0.016	4.584E+06
24 h	4	6.80	6.2	32.3	0.5	1.98	NA	0.0872	0.044	1.073E+06
72 h	1	6.78	3.3	71.9	0.9	3.47	604	0.0486	0.061	1.266E+06
72 h	4	7.66	3.7	171.7	4.3	0.99	966	0.0490	0.039	1.850E+06
1 week	4	8.18	NA	NA	NA	NA	NA	0.0357	NA	2.529E+06
1 week	12	8.41	NA	NA	NA	NA	NA	0.0505	NA	6.678E+06
2 weeks	4	8.21	5.2	138.7	2.1	2.46	NA	0.0609	0.083	1.514E+06
2 weeks	12	8.44	2.9	91.0	1.3	2.79	914	0.0760	0.093	6.097E+05
3 weeks	4	7.08	NA	NA	NA	NA	NA	0.0244	0.005	4.374E+03
3 weeks	12	6.35	NA	NA	NA	NA	NA	0.0411	0.021	6.417E+04
4 weeks	4	7.35	NA	NA	NA	8.50	NA	0.0125	0.018	2.389E+04
4 weeks	12	6.25	4.1	19.6	3.1	5.25	NA	0.0328	0.065	4.213E+04
6 weeks	4	7.65	17.5	182.2	20.6	12.00	NA	0.0457	0.011	1.100E+05
6 weeks	12	7.94	3.4	53.0	12.0	9.50	NA	0.0747	0.071	3.591E+04
7 weeks	4	7.34	3.3	58.6	6.8	6.00	NA	NA	0.014	8.663E+04
7 weeks	12	6.96	NA	NA	NA	32.75	NA	NA	0.082	9.333E+04

*NA, data not available.*

*^*a*^Sample time represents the introduction of water to the soil.*

*^*b*^NO_*x*_ is the sum of NO_*2*_^-^ and NO_*3*_^-^*.

*^*c*^Chl a, chlorophyll a.*

Soil characteristics: Soil temperature was recorded using Maxim Integrated, Inc., Thermochron iButtons as previously utilized in Antarctic soils (Zawar-Reza et al., 2013). iButton temperature loggers were positioned in soil at a distance of 1, 4, and 12 m from the point of water outflow from the diversion at a depth of ∼3–5 cm. Commencing on the 11th of December 2009, the iButtons were set to record temperature every 4 h. The unit located at the 4 m site malfunctioned in the field and data was therefore not available. NO_X_ (NO_3_-+NO_2_-), pH, silicate (SiOH_4_) and orthophosphate (PO_4_), were determined as previously described in Niederberger et al. (2012). Samples for dissolved inorganic carbon (DIC) concentration were gently poured into 20 ml borosilicate scintillation vials, preserved with 200 μl 5% (wt:vol) mercuric chloride and stored in the dark at 4°C until analysis (Sharp et al., 2009). Prior to analysis, samples were brought to room temperature and analyzed using a Monterey Bay Research Institute-clone DIC analyzer with acid-sparging and non-dispersive infrared analysis (LI-COR CO_2_ Analyzer, Model LI-6252) as described previously in Friederich et al. (2002). DIC determinations were made from a single point calibration using certified reference material (A. Dickson at the Oceanic Carbon Dioxide Quality Control, Marine Physical Laboratory at Scripps Institution of Oceanography, UCSD) and were based on triplicate 1.5 ml injections for each determination. NH_4_ was analyzed on 25 ml samples that were filtered through a 25 mm GF/F filter and collected into a 50 ml polypropylene tube. Samples were stored frozen until analysis according to Solorzano (1969) using a 10 cm path-length cell. All results were graphed and any noticeable trends tested via analysis of variance (ANOVA) in Excel (single factor, α = 0.02) at each time period (ignoring location) with two data points.

Biological characteristics and activity: Cell counts, chlorophyll *a* and nitrogenase activity were determined as previously described in Niederberger et al. (2012). Briefly, cell counts were determined using sediments fixed in 2% formalin and stained with DAPI. Chlorophyll *a* was measured via the acidification method of Holm-Hansen et al. (1965). Nitrogenase activity was measured in the field in triplicate using the acetylene (C_2_H_2_) reduction method (Capone, 1993; McKnight et al., 2007). Incorporation of thymidine using primary stocks of [methyl-^3^H] thymidine (60–90.0 Ci/mmol, Perkin-Elmer) were performed in 7 ml plastic scintillation vials according to the protocols of Bell (1993) and Findlay (1993). Small plugs of ∼2 cm^2^ of soil sampled with a sterile 5 ml cut-off syringe were extruded into the vials and slurried with 2 ml of water. Assays used ∼3.8 μCi of [^3^H-methyl] thymidine injections of high specific activity thymidine (73 Ci/mmol, added to 20 nM of final volume) made in 200 μl volumes. Controls included zero time harvested (i.e., injected with radioisotope and immediately fixed with trichloroacetic acid [TCA]) and 3 samples amended with TCA at initiation and incubated over the time course. Following a 30 min incubation period under *in situ* conditions of temperature and light, reactions were terminated and samples filtered onto a 25 mm cellulose acetate filter (0.45 μm) in a Hoefer unit (capturing, collecting and disposing of all filtrate appropriately) rinsing with three washes of cold thymidine. Filters were stored in 15 ml centrifuge vials and frozen for subsequent extraction at the Crary Laboratory, McMurdo Station. Samples were extracted for macromolecules in 5% trichloroacetic acid, centrifuged and the supernatant filtered onto cellulose acetate filters and placed in a scintillation vial overnight to dry and the filter then dissolved with ethyl acetate. Scintillation fluid was added and the samples counted.

DNA extraction, molecular-based analysis and associated statistics: DNA was isolated from soil samples using the PowerSoil^TM^ DNA Isolation Kit (MO BIO) as previously described for MDV soils (Niederberger et al., 2012). Molecular fingerprinting of bacterial and cyanobacteria communities was performed via tRFLP (Niederberger et al., 2012) and ARISA (Wood et al., 2008), respectively, as previously described for Antarctic soils. The eukaryotic tRFLP analyses were undertaken as described by Casamayor et al. (2002) with the exception of using a single restriction endonuclease (MspI; New England BioLabs Inc.) as previously used for Eukaryotic communities in Antarctic soils (Pointing et al., 2009). The archaeal specific primer pair, A751 and UA1406R and thermocycling conditions were used as described by Baker et al. (2003), however, the forward primer was FAM-labeled and *Msp*I used for amplicon digestion. Reactions were of a 25 μl volume containing 14.5 μl PCR Grade Water (MO BIO), 1.5 μl MgCl_2_ (50 mM, Invitrogen), 2.5 μl 10X buffer with MgCl_2_ (Invitrogen), 2.5 μl dNTPs (2 mM, Invitrogen), 1.25 μl each primer (10 μM), 0.5 μl Platinum^®^ *Taq* DNA polymerase (5 U/μl, Invitrogen) and 1 μl of template DNA. For all tRFLP and ARISA assays the terminal-fragments were sized using the MegaBACE system (Amersham) at the Waikato DNA Sequencing Facility (University of Waikato, Hamilton, New Zealand) and fluorescent peak data aligned by the *T-REX* online platform (Culman et al., 2009) and the resulting presence/absence data matrix imported into PRIMER6 (Primer-E Ltd., Plymouth, United Kingdom) for statistical analyses. Principle component analyses (PCA) were undertaken with overlaid percentage similarities (cluster resemblance levels) as detailed in the Primer v6: User Manual/Tutorial (Clarke and Gorley, 2006).

Based on the PCA results, representative samples from *T* = 0, 6 h, 72 h (1 m), 3 weeks (12 m), and 7 week (12 m) were subjected to tag-encoded FLX (Roche) amplicon pyrosequencing of the V1–V3 regions of the 16S and 18S rRNA genes by Research and Testing Laboratories (Lubbock, TX)^1^. Resulting data were then processed using the Quantitative Insights Into Microbial Ecology (QIIME 1) toolkit (Caporaso et al., 2010). In brief, rRNA sequences were quality trimmed (QIIME defaults; >200 bp), split according to barcoded tags and sequences binned into operational taxonomic units (OTU) at 95% similarity. Bacterial taxonomic assignment was undertaken on all quality trimmed 16S rRNA gene sequences using the online RDP classifier tool (at 80% confidence level) and associated RDP release 10.3 database (Cole et al., 2009). Eukaryotic taxonomic assignment was undertaken on a representative sequence from each OTU using the Basic Local Alignment Search Tool (BLAST) within the QIIME toolkit against the SILVA 18S rRNA gene database release 9.1 (Pruesse et al., 2007) as obtained from mothur (Schloss et al., 2009). Library comparisons were performed using the tools within the online RDP pyrosequencing pipeline (RDP release 10.3; Cole et al., 2009). Multidimensional scaling (MDS) plots of pyrosequencing abundance data were performed in PRIMER6. Essentially, the OTU taxonomy abundance table from QIIME was imported in PRIMER6, abundance data transformed (fourth root and presence/absence) and a resemblance matrix (S17 Bray Curtis similarity) constructed for each transformation. CLUSTER and MDS analyses were performed on the matrices and with cluster resemblance levels overlaid. The DIVERSE analysis within PRIMER6 was used to obtain univariate diversity indices: “S” (total OTUs), the number of OTUs in each sample, i.e., OTUs with nonzero counts; “N” (total individuals), the number of individuals in each sample (i.e., number of sequences) and “d” [Margalef’s species richness = (S-1)/Log(N)] which is a measure of the number of OTUs present, making some allowance for the number of individuals. Null hypotheses for community analysis were tested using the Analysis of Similarity (ANOSIM) function in PRIMER6. ANOSIM analysis compares similarities between samples within each group to similarities between groups and generates a value of R between -1 and +1, such that a value of 0 supports the null hypothesis that there were no differences between within-group comparisons and between-group comparisons. Here, we tested the null hypotheses that bacteria, eukaryotic, cyanobacteria and archaeal communities were not different between sampling sites (1, 4, and 12 m) and between sampling times (<1 day, 3 days, 1 week, 2–4 weeks, and 6–7 weeks).

## Results

During the experiment, soil temperatures displayed diurnal cycles between -1°C and +9°C (∼99% of the data points) with extreme highs and lows of 10.5°C and -2.5°C, respectively (Supplementary Figure S1). Samples collected throughout the experiment and associated geochemical and activity data are listed in Table 1. No noticeable trends were observed for pH, NO_x_ (NO_3_^-^ + NO_2_^-^), or chlorophyll *a* during the wetting progression. Orthophosphate and silicate concentrations were not significantly different over time but showed a trend toward increasing concentrations. Similarly an increase in DIC was observed but significance could not be tested due to insufficient data. Ammonium was the only factor that showed significant (*F* = 12.95, *df* = 4, *P* = 0.007544) increase during the first 6 weeks of the experiment (7 week values outliers, due to a larger variance between readings). Microbial activities were also monitored; however, rates were below detection limits (nitrogen fixation ∼ < 0.01–0.03 nmol N/cc/h) or remained stable during the wetting experiment, i.e., thymidine incorporation (Table 1). Similarly, DNA concentrations and cell counts remained stable over the wetting experiment (Table 1).

**Figure 2:**
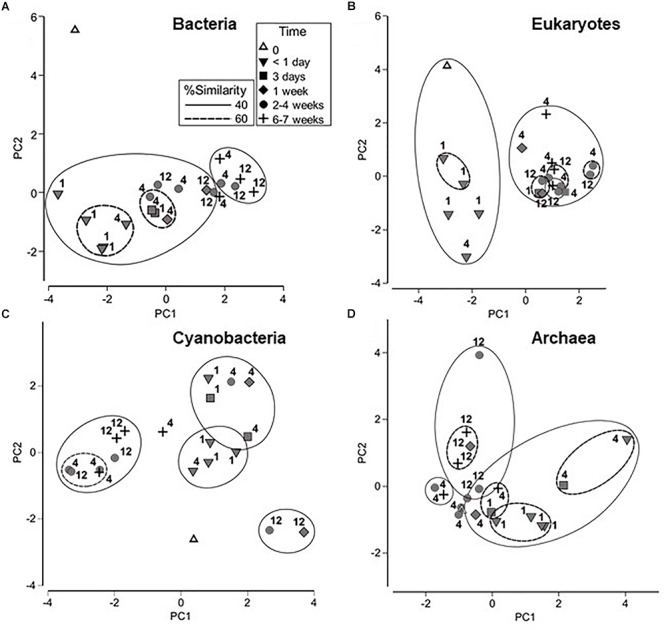
Principle component analyses (PCA) of microbial community fingerprints with overlaid cluster percentage resemblance: **(A)** bacterial tRFLP; **(B)** eukaryotic tRFLP; **(C)** cyanobacterial ARISA, and **(D)** archaeal tRFLP. Numbers signify the location of the sampling site, i.e., 1, 4, or 12 m from the source point of the water outflow.

Bacterial, eukaryotic and archaeal communities were first examined during the course of the wetting experiment using tRFLP-based analyses and the cyanobacteria component using ARISA DNA fingerprinting for a provisional assessment of relative diversity. These data are presented as PCA graphs in Figure 2. It should be noted that 1 m samples were collected only at <1 day and 3 day time points, and the samples from 12 m were collected at 2–4 and 6–7 weeks only. However, the 4 m samples, collected at each time point shown in Figure 2, demonstrate a trend in changing bacterial community structure that is consistent with samples collected at other distances over time. A change in bacterial community structure was evident in <1 day compared to the initial time point, and was likely due to importation from the diverted water source. Continued changes to the community were observed, yielding a final cluster of 40% similarity which contained the majority of the 6 and 7 week samples from 4 and 12 m distances. ANOSIM analysis also indicated that bacterial communities at <1 day were significantly different from those at later time points (*p* < 0.05), and those at 6–7 weeks from 4 and 12 m distances were significantly different from bacterial communities at 2–4 weeks from these same sites (Supplementary Table S1).

Eukaryotic profiling demonstrated the presence of two major groups (at 40% similarity levels) encompassing soil communities collected <1 day from the commencement of wetting with the remainder of the samples forming a separate isolated cluster. Statistical analysis using ANOSIM supported these results, showing that the eukaryotic community in samples collected <1 day were significantly different from those collected at later time points (*p* < 0.05; Supplementary Table S1). Eukaryotic communities collected at the 1 m sampling site were also significantly different from those at 4 and 12 m (*p* < 0.05).

The cyanobacteria communities showed a transition during the wetting experiment from a distinct time zero profile to a cluster (at 40% similarity levels) which included the bulk of samples from weeks 2–7. ANOSIM analysis indicated that the cyanobacterial community in samples collected at < 1 day after initiation of the experiment was significantly different (*p* < 0.05) from those collected at 2–4 and 6–7 weeks (Supplementary Table S1). In contrast to the bacterial and eukaryotic communities, however, there were no significant differences in cyanobacteria communities between sampling sites.

Trends were not as apparent for the archaeal communities. However, ANOSIM analysis showed that the archaeal community at 1 m was significantly different from the community at 12 m (*p* < 0.05) and that the samples collected at < 1 day were significantly different from samples collected at 6–7 weeks (*p* < 0.05) (Supplementary Table S1).

Representative samples were chosen based on the PCA profiles (Figure 2) and both bacterial and eukaryotic rRNA genes were pyrosequenced to provide an in-depth analysis of changes in the community composition and structure. Supplementary Table S2 lists the OTU totals, the number of OTUs and species richness for each sample. Bacterial communities displayed a steady increase in the total observed OTUs over the course of the experiment, from 60 to 132 with a corresponding increase in species richness. These trends were not mirrored in the eukaryotic data. Rarefaction analysis (Supplementary Figure S2) suggests that bacterial communities may have been under-sampled, however, analysis of eukaryotic OTUs indicated that the majority of the community was captured in the pyrosequencing effort.

**Figure 3:**
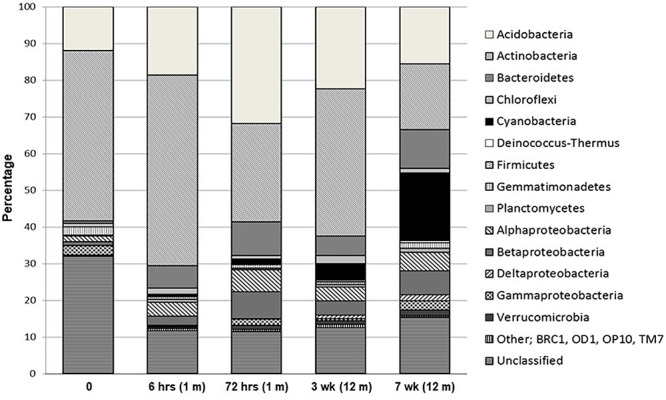
Percentage distribution of bacterial phylogenetic groups from pyrosequenced 16S rRNA genes.

Percentage distribution of bacterial phylogenetic groups from pyrosequenced 16S rRNA genes is presented in Figure 3. A large proportion of sequences were unclassified, ranging from 11 to 32% (RDP release 10.3). Obvious trends over the time-course of wetting included universally high percentages of Acidobacteria and Actinobacteria at all sites, making up 12–32% and 18–52%, respectively, in all samples. With the exception of the final sample at 7 weeks, the Acidobacteria and Actinobacteria combined made up over 50% of each community from all time points. Acidobacteria were dominated by the Gp4 phylogenetic group while members of the Rubrobacterineae were the most dominant type of Actinobacteria for all sites, but decreased during the wetting experiment from 38% at 0 h to 9% at 7 weeks. In contrast, Cyanobacterial signatures were below 1% of the library at time zero and remained at < 5% of the total sequences until week 7, where it made up 19% of the library and was the dominant group. Bacteroidetes comprised less than 1% of the total community at time zero but increased to ∼ 11% at 7 weeks. These sequences consisted almost exclusively of members of the Flavobacteria and the Sphingobacteria. Notably, Flavobacteria were not detected in the time zero sample but were present in all other samples. The Deinococcus–Thermus phylogenetic group were absent at time zero and remained low (<1%) throughout the experiment consisting almost exclusively of the genera *Deinococcus* and *Truepera*. Members of the Firmicutes (dominated by members of the Clostridiales), Gemmatimonadetes and Planctomycetes constituted minor members (<2%) of the communities. The proteobacteria increased from <2.5% of total community to ∼13%, primarily due to substantial increases in the alpha- and beta-proteobacteria population. The gamma-proteobacteria, dominated by members of the Xanthomoadaceae, remained consistently below 2.5% for all sites. Verrucomicrobia sequences steadily increased from ∼0.2 to 1.5% throughout the wetting period. MDS and cluster analyses of the phylogenetic abundance data from the pyrosequencing effort (Supplementary Figure S3) reflected the results of PCA using the ARISA and tRFLP data, whereby the time zero sample was the most divergent sample between all sites. Moreover, results were identical when the data were treated as presence/absence (results not shown). Time zero community assemblages were then compared to those from all other time points to identify signatures that contributed most to the dissimilarity. The most significant differences in sequence abundance between time zero and all other time points are listed in Supplementary Table S3. The major differences (significance ≤ 1.77E-26) included an increase in sequences from higher levels of Cyanobacteria (Family I, GpI), Comamonadaceae, Flavobacteria (*Flavobacterium*), Chloroplast sequences, *Polaromonas* and Xanthomonadales in the T ≠ 0 soils.

**Figure 4:**
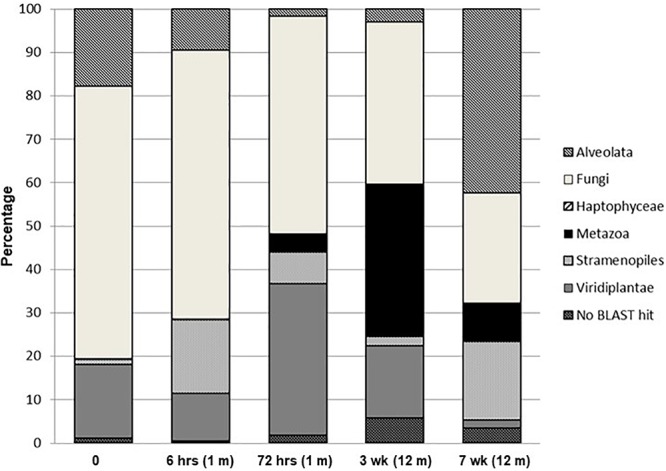
Percentage distribution of eukaryotic phylogenetic groups from pyrosequenced 18S rRNA genes.

Changes in the percent distribution of eukaryotic phylogenetic groups from pyrosequenced 18S rRNA genes are presented in Figure 4. Overall, with the exception of the final week 7 sampling time, communities were dominated by Fungi, which decreased from ∼62% of the eukaryotic signatures at time zero to ∼25% at 7 weeks. The composite sample collected at time zero was dominated (42% of total community) by *Tilletiopsis*-related fungal sequences, Sporobolomyces were the second most dominant group in the time zero soils, comprising ∼13% of the total community, and were the dominant fungal signature for the remainder of the time points. The final week 7 soil sample was dominated by members of the Alveolata (42%) with the most dominant signature (∼30% of the total eukaryotic signatures) related to the ciliate, *Halteria* (not exceeding 8% in each of the remaining community profiles). The algal division of the Haptophyceae encompassed a minor presence (<0.15%) and were only detected in the time zero and 12 h sampling points. Metazoa exhibited an increase over the wetting period, being below detection levels at time zero, 0.03% at 6 h, 4% at 72 h, 35% at 3 weeks and 9% after 7 weeks. This phylogenetic group consisted almost solely of nematode signatures related to *Paracanthonchus*. The abundance of Stramenopiles over the wetting experiment varied and was dominated by signatures related to the bacteriovorus protist *Paraphysomonas* and the chrysophyte, *Chrysosaccus*. These two signatures increased during the experiment from <1% at time zero to 13% at 7 weeks. Diatoms belonging to the genus *Navicula* were only detected in the final sampling time point (comprising ∼4% the total community). The contribution from Viridiplantae signatures varied during the wetting experiment with a maximum of 35% after 72 h and a minimum at 7 weeks (∼2%), and were almost completely dominated by the green algae *Coenocystis*. Unidentified eukaryotic sequences for each sample (no BLAST hits) ranged between approximately 0.5 and 6% of the total signatures. MDS and cluster analyses of the phylogenetic pyrosequence abundance data (Supplementary Figure S4) for all sampling points reflected the PCA analyses, i.e., having a highly distinct time zero sample, with earlier samples (6 and 72 h) and later samples (3 and 7 weeks) forming distance clusters. Similar MDS and cluster results were obtained when data were treated as presence/absence (results not shown).

## Discussion

Our objectives were to simulate as natural a wetting event as possible to allow the resident community to respond not only to the new environmental conditions but also to the introduction of any hydrobiology. Results of this manipulation demonstrated rapid and significant changes in bacterial community structure and soil parameters during a 7 weeks wetting experiment of archetypal arid MDV soils. This work contributes to a growing body of evidence that suggests MDV soil communities may respond rapidly to changes in environmental conditions, over a matter of weeks (Schwartz et al., 2014; Van Horn et al., 2014; Buelow et al., 2016). Due to multiple silting, as well as freezing events inherent in this natural system, the flow of water to soils was irregular. The flow also varied over distance, with greater flow at sites closer to the input, while more distant sites were only wetted. These changes may therefore occur within shorter timeframes if a constant water source was presented. Moreover, as these communities do not inhabit areas where rapid responses might be expected (e.g., previously wetted stream/lake associated soils), this sensitivity to environmental changes was even more unexpected. It could be argued that changes were largely due to input of biomass from the diverted water source. However, proxies for biomass such as cell counts and chlorophyll *a* concentrations did not increase significantly over the course of the experiment, suggesting that addition of biomass alone from the diverted water source was minimal (Table 1). Shifts in composition toward more “wet-specific” signatures, on the other hand, indicated recruitment of species that may have rapidly colonized the arid soil community, and/or a reactivation of cryptobiotic species within the arid sediments upon addition of water (McKnight et al., 2007).

Temperatures during the experiment followed solar radiance cycles with minima dipping below 0°C and maxima within ranges expected for summer streams (8–10°C; McKnight et al., 2007). The time-zero, composite soil sample resembled a classic arid MDV soil by having a near-neutral pH (Lee et al., 2012), ammonium, chlorophyll *a* and orthophosphate concentrations within, or below ranges previously reported for MDV soils and stream waters (McKnight et al., 1998, 2007; Niederberger et al., 2008; Zeglin et al., 2011; Bottos et al., 2014a) and a microbial community dominated by Fungi and Actinobacteria (Lee et al., 2012). Actinobacteria are commonly present as dominant members of arid MDV soils (Smith et al., 2006; Niederberger et al., 2008; Pointing et al., 2009; Lee et al., 2012; Bottos et al., 2014b) and therefore their perceived decrease during the wetting experiment was unsurprising. Although nitrogen fixation was below detection levels and bacterial activity varied but did not increase throughout the experiment (Table 1), changes in soil water characteristics during the wetting experiment reflect the acceleration of microbial activities either *in situ* or in the water input over time. This included a doubling of DIC during the first 2 weeks of the experiment and a significant ∼10–60-fold increase in ammonium over the 6–7 week period. DIC accumulation is typically associated with increased CO_2_ production due to microbial respiration and mineralization of organic carbon (Moyano et al., 2013). Ammonium is also an indicator of biological activity in the MDV with high concentrations of ammonium (Barrett et al., 2002) measured in sites of high biodiversity and biological abundance. The natural deposition of ammonium in the MDV is negligible and it is therefore hypothesized to be derived from the biological mineralization of organic N (Barrett et al., 2002).

As may have been predicted, the most significant bacterial change in community structure during the experiment was the enrichment of cyanobacteria. Additional signatures related to organisms adapted to aquatic conditions also increased over the experiment including members of the chloroflexi, the swimming ciliate, *Halteria*, freshwater protists *Paraphysomonas* and *Chrysosaccus* and the aquatic photosynthetic diatom *Navicula*. Furthermore, signatures commonly found in wetted MDV soils increased in frequency during the experiment including Bacteroidetes (in particular members of the Flavobacteria) and members of the alpha- beta- and delta-proteobacteria. Bacteroidetes have previously been shown to be the most abundant heterotrophic taxa in ephemerally wetted MDV microbial mats and sediments (Zeglin et al., 2011; Stanish et al., 2013; Van Horn et al., 2016), likely supported by their ability to degrade various organic compounds. Flavobacteria have also been identified in arid mineral soils (Smith et al., 2006), suggesting that increases in Flavobacteria may be due in part to growth of a resident population after water and nutrient input. Our results further support the study by Stanish et al. (2013) whereby the authors detected large concentrations of both alpha- and beta-proteobacteria signatures in wetted MDV soils. These signatures became increasingly common during our experiment in the ephemerally wetted soils. In particular, *Polaromonas* of the Comamonadaceae significantly increased over the course of the experiment. This alphaproteobacteria is common in wetted MDV soils but is also widely distributed as dormant cells (Darcy et al., 2011) and, similar to Flavobacteria, may represent a member of the resident community within the arid sediments, reactivated upon addition of water. In addition, the community now included taxa that are associated with “high-productivity” MDV communities. The increase in cyanobacteria, Verrucomicrobia, alpha- and beta-proteobacteria and Xanthomonadales are consistent with results from an earlier molecular clone-library based study comparing low and high-productivity soil sites in the Luther Vale region of Northern Victoria Land (Niederberger et al., 2008). In this study, signatures of the Verrucomicrobia, Betaproteobacteria, and the genus *Xanthomonas* of the Gammaproteobacteria were found exclusively in wet “high-productivity” soils. In addition, live nematodes were detected in both low and high productivity sites (Niederberger et al., 2008) affirming that water does not appear to be the primary limiting factor for the presence of nematodes in MDV soil habitats (Virginia and Wall, 1999). However, as witnessed during our experiment, an increase in nematode signatures further suggests that a wetted environment can select for, and may be preferred by these organisms (Adams et al., 2014), while other factors likely shape nematode species assemblages (Poage et al., 2008). Taken together, the results show that within a 7 week time-period, arid MDV soil communities have the potential to undertake dramatic shifts in community structure thereby adapting to local environmental conditions. Interestingly, some of most significant wet-specific signatures such as *Flavobacterium*, *Polaromonas*, Oxalobacteraceae (in particular *Duganella*), Chloroplast signatures, *Chrysosaccus*, and *Navicula*, were not detected at time-zero suggesting that these organisms likely colonized soils via transportation to the site by the water source. However, at least for the bacterial signatures, rarefaction curves did not reach saturation, suggesting this observation potentially related to an inadequate sample size.

Shifts in community composition for all three domains of life were observed within the 7 week time period. Of particular interest are the archaea. Often overlooked or undetected [which may in-part be linked to historical PCR-based biases (Baker et al., 2003)], archaea have been typically absent in ecological studies of MDV soils (de la Torre et al., 2003; Niederberger et al., 2008; Pointing et al., 2009; Lee et al., 2012). However, in addition to our study, recent work indicates that archaea may be more common in MDV soils than previously reported (Ayton et al., 2010; Richter et al., 2014). Moreover, archaeal species richness may be positively correlated with soil water content, hence water may be a chief driver of archaeal community richness (Richter et al., 2014). Nevertheless, more work is needed to ascertain the ecological role of archaea in MDV soils.

Previous studies have observed increased microbial diversity in both hot and cold desert soils with higher water content (Pointing et al., 2007; Aislabie and Bowman, 2010), however it is not yet fully clear if this trend holds true in ephemerally wetted soils which experience cycles of wetting and drying. Previous reports indicated that increases in bacterial community richness are not correlated to sediment water content in the MDV (Zeglin et al., 2011), but instead may be a response to carbon content (Geyer et al., 2013). Here, a steady increase in both bacterial species richness and DIC over the wetting period was witnessed during our experiment, with an approximate two fold increase in species richness in 7 weeks. This increase in species richness may represent the early phase of transition to a high productivity microbial mat, whereby the initial recruitment and colonization of a range of wet-adapted species is followed by establishment of a subset of the community.

The changes in community composition and species richness observed in this study may also reflect the awakening of cryptobiotic species. Van Horn et al. (2014) demonstrated the effects of resource addition in the form of water and organic matter to MDV soils, resulting in rapid (30 days) and significant changes in activity and composition of microbial communities. McKnight et al. (2007) also demonstrated a rapid increase in cyanobacterial mat biomass and biological activity after introduction of water to a relic stream, showing that biological communities maintain the potential to respond quickly to renewed flow (McKnight et al., 2007). While these experiments differed substantially from the wetting experiment described here, results of these and other studies (e.g., Schwartz et al., 2014; Aanderud et al., 2018) demonstrate that endemic communities in the MDV can respond within timeframes considerably shorter than previously hypothesized, thereby challenging long-held perceptions regarding the MDV of extremely slow response rates (Burkins et al., 2001; Elberling et al., 2006; Barrett et al., 2007).

Due to the relative simplicity of MDV ecosystems, it is postulated that these habitats may be particularly sensitive to climate change (Hogg et al., 2006; Cary et al., 2010; Nielsen and Wall, 2013). Changes to these unique and threatened communities may represent important early-warning indicators of ecological shifts (Bottos et al., 2014a). Therefore, *in situ* manipulative field-based studies such as the one conducted here have important implications for understanding how microbial communities may respond to these future predictions. Current predictions project that Antarctic soils will become warmer and wetter (Bracegirdle et al., 2008) leading to cascading changes in hydrology that will ultimately affect the availability and distribution of liquid water in the MDV (Fountain et al., 2014). Results of this manipulation suggest that MDV soil communities are strikingly responsive to changes in water availability. This work is consistent with current predictions that these communities are highly sensitive to climate change scenarios, and further support the notion that changes in microbial community structure and associated biochemical cycling may occur much more rapidly than predicted.

## Author Contributions

The project was designed and implemented by SC. Fieldwork, laboratory, and data analysis were conducted by TN, JS, TG, AP, EB, DC, KC, and SC. EB remained in the field during the entire season to service the experiments. The manuscript was written and edited by TN, SC, KC, EB, DC, and EC.

## Conflict of Interest Statement

The authors declare that the research was conducted in the absence of any commercial or financial relationships that could be construed as a potential conflict of interest.

## Footnotes

1 www.researchandtesting.com
