# Supplementary material for: Rapid Microbial Dynamics in Response to an Induced Wetting Event in Antarctic Dry Valley Soils
Source: Front Microbiol. 2019 Apr 4;10:621. doi: 10.3389/fmicb.2019.00621 (PMC6458288; doi:10.3389/fmicb.2019.00621)
Supplement: Supplementary file 1 [file Data_Sheet_1.docx]

Supplementary Material

**Rapid microbial dynamics in response to an induced wetting event in Antarctic Dry Valley soils**

**Thomas D. Niederberger, Jill A. Sohm, Troy Gunderson, Alex Parker, Eric M. Bottos, Kathryn J. Coyne, Douglas G. Capone, Edward J. Carpenter, S. Craig Cary^*^**

- **Correspondence:** caryc@udel.edu

**Supplemental Figure S1** – Soil temperatures at 1 and 12 meters.


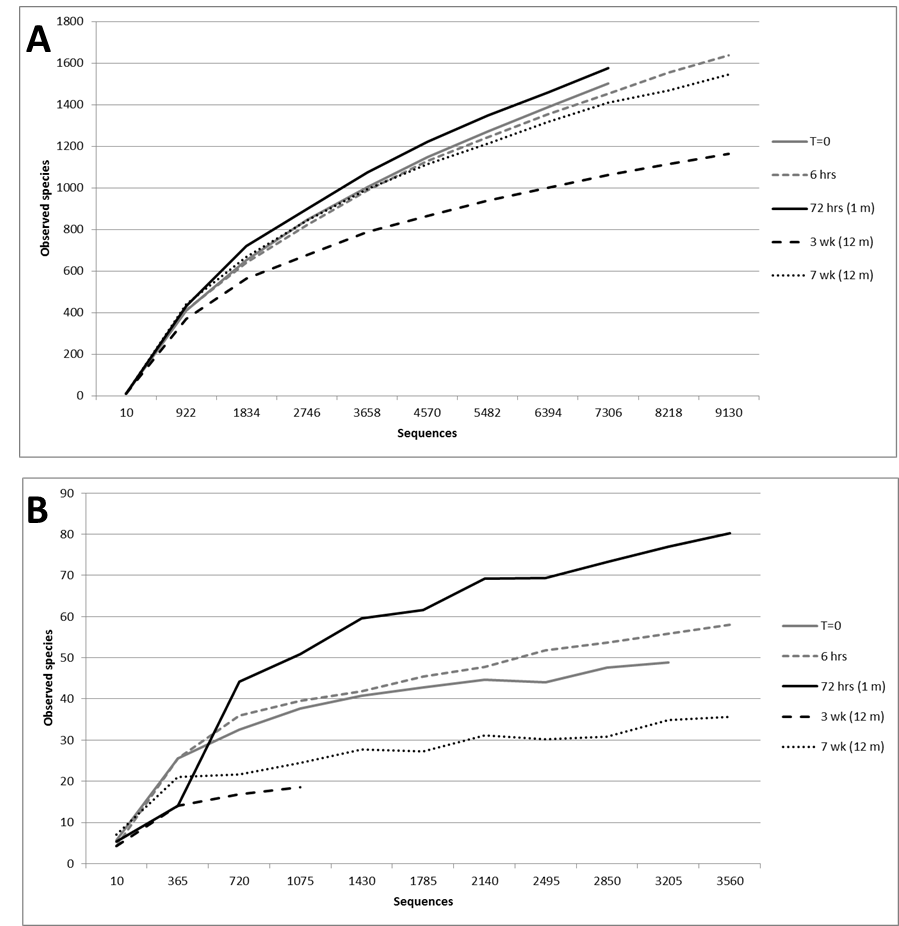
**Supplemental Figure S2** – Rarefaction analysis. A, bacterial community sequencing. B, eukaryotic communities sequencing


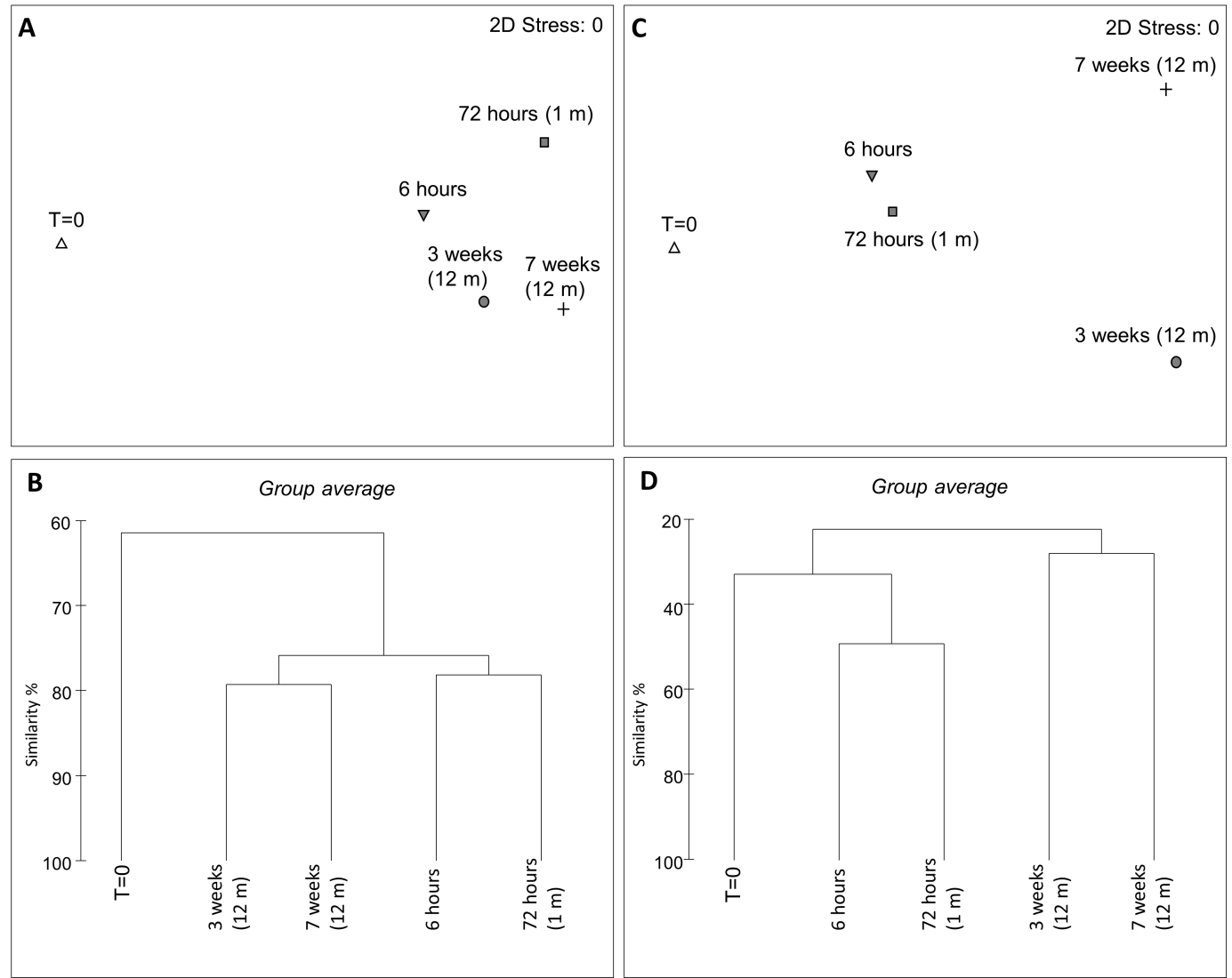


**Supplementary Fig. S3** - Multidimensional scaling (MDS) plots of pyrosequencing abundance data for (A) bacterial and (C) eukaryotic sequences and associated cluster plots (resemblance:S17 Bray Curtis similarity) for (B) bacterial and (D) eukaryotic sequences.

| **Sample** | **S**  **(total species, OTUs at 95%)** | **N**  **(number of sequences)** | **d**  **(species richness)** |
| --- | --- | --- | --- |
| **Bacteria** |  |  |  |
| T = 0 | 60 | 8213 | 6.546 |
| 6 hours | 88 | 9137 | 9.539 |
| 72 hours (1 m) | 109 | 7575 | 12.09 |
| 3 weeks (12 m) | 105 | 10633 | 11.22 |
| 7 weeks (12 m) | 132 | 10361 | 14.17 |
| **Eukaryotes** |  |  |  |
| T = 0 | 50 | 3452 | 6.015 |
| 6 hours | 58 | 3563 | 6.97 |
| 72 hours (1 m) | 92 | 5171 | 10.64 |
| 3 weeks (12 m) | 19 | 1126 | 2.562 |
| 7 weeks (12 m) | 45 | 6335 | 5.026 |

**Supplemental Table S2.** Statistics from the sequencing of both bacterial and eukaryotic communities.

| Rank | Name | T = 0 | % of total seqs. | T ≠ 0 | % of total seqs. | Significance |
| --- | --- | --- | --- | --- | --- | --- |
| phylum | Cyanobacteria | 4 | 0.049 | 2542 | 6.74 | 8.36E-209 |
| family | Comamonadaceae | 2 | 0.024 | 784 | 2.08 | 1.32E-63 |
| order | Flavobacteriales | 0 | 0 | 645 | 1.71 | 1.04E-55 |
| class | Flavobacteria | 0 | 0 | 645 | 1.71 | 1.04E-55 |
| family | Flavobacteriaceae | 0 | 0 | 566 | 1.50 | 5.97E-49 |
| genus | *Flavobacterium* | 0 | 0 | 520 | 1.38 | 5.16E-45 |
| family | Family I | 3 | 0.037 | 414 | 1.10 | 4.35E-31 |
| genus | GpI | 3 | 0.037 | 414 | 1.10 | 4.35E-31 |
| family | Chloroplast sequences | 0 | 0 | 326 | 0.86 | 2.07E-28 |
| genus | *Polaromonas* | 0 | 0 | 317 | 0.84 | 1.22E-27 |
| order | Xanthomonadales | 4 | 0.049 | 375 | 0.99 | 1.22E-26 |
| family | Xanthomonadaceae | 4 | 0.049 | 373 | 0.99 | 1.77E-26 |
| family | Oxalobacteraceae | 0 | 0 | 287 | 0.76 | 4.50E-25 |
| family | Rhodobacteraceae | 1 | 0.012 | 280 | 0.74 | 9.17E-23 |
| order | Rhodobacterales | 1 | 0.012 | 280 | 0.74 | 9.17E-23 |
| family | Sphingomonadaceae | 3 | 0.037 | 296 | 0.79 | 2.04E-21 |
| family | Caulobacteraceae | 0 | 0 | 233 | 0.62 | 1.88E-20 |
| order | Caulobacterales | 0 | 0 | 233 | 0.62 | 1.88E-20 |
| genus | *Duganella* | 0 | 0 | 196 | 0.52 | 2.76E-17 |
| genus | *Flavisolibacter* | 1 | 0.012 | 200 | 0.53 | 4.64E-16 |
| suborder | Micrococcineae | 2 | 0.024352 | 205 | 0.54368 | 3.38E-15 |

**Supplemental Table S3.** Library comparison (myRDP) of T= 0 bacterial sequences (total sequences = 8213) vs. T ≠ 0 combined bacterial sequences (total sequences = 37706). Only the most significant (<6.00E-14) differences are presented
