# Supplementary material for: Rapid Microbial Dynamics in Response to an Induced Wetting Event in Antarctic Dry Valley Soils
Source: Front Microbiol. 2019 Apr 4;10:621. doi: 10.3389/fmicb.2019.00621 (PMC6458288; doi:10.3389/fmicb.2019.00621)
Supplement: Supplementary file 2 [file Data_Sheet_2.docx]

Supplementary Table S1. ANOSIM results.

ANOSIM results yielding significant differences between cyanobacteria assemblages

| Comparison (factor) | R statistic | Significance level (%) |
| --- | --- | --- |
| Global test (Time) | 0.274 | 1.8 |
| Pairwise tests (Time) |  |  |
| <1 day vs. 2-4 weeks | 0.468 | 2.4 |
| <1 day vs. 6-7 weeks | 0.647 | 0.8 |

ANOSIM results yielding significant differences between bacteria assemblages

| Comparison (factor) | | R statistic | | Significance level (%) | |
| --- | --- | --- | --- | --- | --- |
| Global test (Site) | | 0.286 | | 0.2 |  |
| Pairwise tests (Site) | |  | |  |  |
| 1 m vs. 12 m | | 0.683 | | 0.2 |  |
| 4 m vs. 12 m | | 0.192 | | 4.3 |  |
|  | |  | |  |  |
| Global test (Time) | | 0.420 | | 0.2 |  |
| Pairwise tests (Time) | |  | |  |  |
| <1 day vs. 3 days | | 0.582 | | 4.8 |  |
| <1 day vs. 1 week | | 0.818 | | 4.8 |  |
| <1 day vs. 2-4 weeks | | 0.435 | | 0.9 |  |
| <1 day vs. 6-7 weeks | | 0.900 | | 0.8 |  |
| 2-4 weeks vs. 6-7 weeks | | 0.542 | | 0.5 |  |
|  |  | |  |  |  |

ANOSIM results yielding significant differences between Archaea assemblages. NS, non-significant

| Comparison (factor) | R statistic | Significance level (%) | |
| --- | --- | --- | --- |
| Global test (Site) | 0.168 | 1.8 |  |
| Pairwise tests (Site) |  |  |  |
| 1 m vs. 12 m | 0.683 | 0.6 |  |
|  |  |  |  |
| Global test (Time) | 0.106 | 17.9 (NS) |  |
| Pairwise tests (Time) |  |  |  |
| <1 day vs. 6-7 weeks | 0.672 | 0.8 |  |

Supplementary Table S1 (continued)

ANOSIM results yielding significant differences between eukaryotic assemblages

| Comparison (factor) | R statistic | Significance level (%) | |
| --- | --- | --- | --- |
| Global test (Site) | 0.255 | 0.2 |  |
| Pairwise tests (Site) |  |  |  |
| 1 m vs. 4 m | 0.231 | 2.7 |  |
| 1 m vs. 12 m | 0.548 | 0.2 |  |
|  |  |  |  |
| Global test (Time) | 0.561 | 0.1 |  |
| Pairwise tests (Time) |  |  |  |
| <1 day vs. 3 days | 0.691 | 4.8 |  |
| <1day vs. 1 week | 0.727 | 4.8 |  |
| <1day vs. 2-4 weeks | 0.725 | 0.2 |  |
| <1day vs. 6-7 weeks | 0.919 | 0.8 |  |
